# Repeated Digitized Assessment of Risk and Symptom Profiles During Inpatient Treatment of Affective Disorder: Observational Study

**DOI:** 10.2196/24066

**Published:** 2020-12-01

**Authors:** Maike Frederike Richter, Michael Storck, Rogério Blitz, Janik Goltermann, Juliana Seipp, Udo Dannlowski, Bernhard T Baune, Martin Dugas, Nils Opel

**Affiliations:** 1 Department of Psychiatry University of Münster Münster Germany; 2 Institute of Medical Informatics University of Münster Münster Germany; 3 Department of Psychiatry Melbourne Medical School The University of Melbourne Melbourne Australia; 4 The Florey Institute of Neuroscience and Mental Health The University of Melbourne Parkville Melbourne Australia; 5 Interdisciplinary Centre for Clinical Research Münster University of Münster Münster Germany

**Keywords:** affective disorders, digital data collection, psychiatry, P4 medicine

## Abstract

**Background:**

Predictive models have revealed promising results for the individual prognosis of treatment response and relapse risk as well as for differential diagnosis in affective disorders. Yet, in order to translate personalized predictive modeling from research contexts to psychiatric clinical routine, standardized collection of information of sufficient detail and temporal resolution in day-to-day clinical care is needed. Digital collection of self-report measures by patients is a time- and cost-efficient approach to gain such data throughout treatment.

**Objective:**

The objective of this study was to investigate whether patients with severe affective disorders were willing and able to participate in such efforts, whether the feasibility of such systems might vary depending on individual patient characteristics, and if digitally acquired assessments were of sufficient diagnostic validity.

**Methods:**

We implemented a system for longitudinal digital collection of risk and symptom profiles based on repeated self-reports via tablet computers throughout inpatient treatment of affective disorders at the Department of Psychiatry at the University of Münster. Tablet-handling competency and the speed of data entry were assessed. Depression severity was additionally assessed by a clinical interviewer at baseline and before discharge.

**Results:**

Of 364 affective disorder patients who were approached, 242 (66.5%) participated in the study; 88.8% of participants (215/242) were diagnosed with major depressive disorder, and 27 (11.2%) had bipolar disorder. During the duration of inpatient treatment, 79% of expected assessments were completed, with an average of 4 completed assessments per participant; 4 participants (4/242, 1.6%) dropped out of the study prematurely. During data entry, 89.3% of participants (216/242) did not require additional support. Needing support with tablet handling and slower data entry pace were predicted by older age, whereas depression severity at baseline did not influence these measures. Patient self-reporting of depression severity showed high agreement with standardized external assessments by a clinical interviewer.

**Conclusions:**

Our results indicate that digital collection of self-report measures is a feasible, accessible, and valid method for longitudinal data collection in psychiatric routine, which will eventually facilitate the identification of individual risk and resilience factors for affective disorders and pave the way toward personalized psychiatric care.

## Introduction

In what has become known as *P4* or *precision medicine* [1,2] a major goal of medical research and applied health care is the evolution from a reactive treatment approach toward medical care that is predictive, preventative, personalized, and participatory.

This approach is particularly relevant in the field of psychiatry, as the imprecise nature of psychiatric nosology, in part due to the heterogeneity of clinical populations, complicates the identification of vulnerable groups and effective treatments [3,4]. Affective disorders such as major depressive disorder exemplify this problem. Only approximately one-third of patients with moderate to severe depression respond to the first treatment attempt with medication [5,6]. This leads to a prolonged illness duration for nonresponders, which is associated with worse overall health outcomes and significantly higher costs to the health care system [7,8]. Precision psychiatry could help alleviate this problem by predicting (P1) the occurrence of depression as well as individual disease course and preventing (P2) unfavorable outcomes such as chronification and suicide by personalizing (P3) treatment plans according to individual risk and resilience factors.

Preliminary attempts have been made to achieve the prediction of disease course and treatment outcome of major depressive disorder through the use of predictive modeling approaches [9-13]. These are a first step toward the identification of biomarkers for depression, which may ultimately inform clinicians who is at risk for relapse or a particularly severe outcome and would benefit from more invasive interventions such as electroconvulsive therapy [14]. However, previous work relies on extremely homogeneous study populations that are carefully selected according to strict inclusion and exclusion criteria. Any predictive models using such data are of little value when findings are to be generalized to clinical reality—highly diverse inpatient populations [15,16]. In contrast to the aforementioned data from homogeneous, well-characterized study samples, data that are routinely gathered in clinical practice are highly heterogeneous, unvalidated, and often not standardized or are inaccessible for predictive analysis [17].

In order to achieve the long-term aims of precision medicine in major depressive disorder treatment, the implementation of a standardized data collection routine in naturalistic environments from real clinical populations is needed. While high temporal resolution data on sleep and activity levels can be tracked with smartphone- or wearable technology–based solutions [18], differentiated data on the patients’ mood or affective state—the core features of major depressive disorder diagnosis—are needed for precise and valid models of affective disorders that accurately reflect the disease and treatment course. As this information needs to be provided by the patients themselves, much emphasis needs to be put on the participatory (P4) aspect of precision medicine in psychiatry. Patients thus need to be engaged and participate actively by contributing either self-report measures at regular intervals or by participating in clinical interviews or ratings. As external assessments are time-intensive and require clinical training, the use of self-rating scales, which can be completed by patients independent from the presence of a researcher or clinician, might be preferable. Recent evidence suggests reasonably high agreement when comparing patient self-reports with diagnostic clinical interviews [19], which supports their use in clinical practice. Previous studies also found the incorporation of self-report measures of symptom severity into routine care to foster engagement between patients and health care professionals and enhance care delivery in various fields of medicine [20]. In psychiatric populations with affective disorders however, questions can be raised as to the patients’ ability and motivation to provide such data, considering the lack of energy as well as cognitive impairments that define major depressive disorder during an incapacitating episode requiring inpatient treatment [21,22]. The difficulty of recruiting depressed patients for randomized controlled trials has been well documented [23], although some investigations revealed that patients did report positive attitudes toward research participation when they felt they were contributing meaningfully to the advancement of major depressive disorder treatments [24]. It remains unclear how the collection of standardized patient reports throughout the treatment course would compare in inclusion rate to the usually much more time-intensive and elaborate study protocol of a randomized controlled trial. It remains equally unknown whether certain patient subgroups may be systematically less willing or able to provide such data regularly, either due to their symptom severity or other disease-specific or sociodemographic factors, which may constitute exclusion criteria in randomized controlled trials.

Another point to consider when striving to make health care truly participatory is that assessments should preferably be collected in a digital format, as digitization allows for quick data analysis and, ideally, feedback for patients on their personal outcomes [15,25]. In general, digital solutions outperform paper-and-pencil questionnaires in practicality, acceptability, and completeness of data across studies in different fields [26-28]. Digital data collection with tablet computers, specifically, is well accepted among psychiatric patients [29]. However, previous investigations with patient reports in psychiatry only included a single assessment or pre–post comparisons as opposed to tracking individual symptom levels throughout the duration of their hospitalization [27]. The feasibility and acceptability of such a study protocol in psychiatric populations remains therefore hitherto unclear.

We established a system of longitudinal digital data collection that gives patients the opportunity to participate actively in providing data concerning their mood and symptom levels throughout the course of their inpatient treatment for an affective disorder via tablet computers. This study assessed whether affective disorder inpatients are willing and able to participate in repeated digital data entry throughout the treatment course. We additionally examined whether age, gender, symptom severity, and global functioning systematically co-vary with the feasibility and acceptability of such research efforts in clinical populations with affective disorders. We furthermore aimed to validate self-report measures of depression severity with the use of an external assessment performed by a clinical interviewer.

## Methods

### Sample

A total of 364 psychiatric patients who were recently admitted to the inpatient service of the Department of Psychiatry, University of Münster were approached during the assessment period from March 2019 to March 2020. Patients who were admitted to the closed ward could not be assessed. Patients who were admitted and discharged over the course of one weekend could equally not be assessed due to the study design, which required presence of study personnel to assist in tablet handling. Criteria for initial eligibility were therefore the admission to any of the open inpatient services of the hospital, a treatment duration of more than 3 days, and the diagnosis of any affective disorder (International Statistical Classification of Diseases, Tenth Revision codes F30.0 through F39.9) at the time of admission. In order to be included as an active participant, patients needed to be sufficiently mentally stable, cognitively able, and proficient in reading and writing German to fill in questionnaires. Due to the naturalistic setting of this investigation, inclusion criteria were intentionally kept as broad as possible in order to achieve the best possible representation of the true population seeking psychiatric inpatient treatment. The study was approved by the local institutional review board and written informed consent was obtained before participation. Patients did not receive compensation for their participation.

### Procedure

Patients with the appropriate diagnoses were identified with a patient recruitment system [30] within the electronic health record based on the diagnosis entered by the treating clinician after an initial examination and diagnostic exploration on the day of admission to the ward. The clinical team approved research participation for all included patients and could dissent to participation when patients were not suitable due to their mental and cognitive symptom severity or insufficient language skills. All other potential participants were approached in hospital within 1 week of beginning their inpatient treatment. They were informed about the study and invited to participate for the duration of their stay at several regular intervals. Participants were informed about the possibility to participate in a scientific study and that the aims of the study were, first, the investigation of potential changes in affective state over the course of treatment as well as the identification of risk or resilience factors, which may influence treatment response. Second, patients were informed that the clinical team would have access to the collected measures and could make use of them as an additional source of information during clinical decision making. A reason for exclusion was recorded for patients who declined regular participation or were excluded by clinicians.

Upon agreeing to participate, patients were, first, given a tablet-based battery of baseline questionnaires, including questions regarding sociodemographic variables, family and own mental health history, childhood trauma, personality style as well as symptom-specific self-report measures. External assessments of depressive symptoms and global functioning were additionally conducted by the researcher at baseline. Participants then provided data on their symptom severity every other week. Immediately before being discharged, they completed selected questionnaires one additional time and were once again assessed externally on their depressive symptoms and global functioning. Please refer to Multimedia Appendix 1 for additional details about the specific measures included in each assessment battery. A researcher was present during data entry, to distribute the tablets and assist patients in case of uncertainty or problems with handling the equipment. The amount of assistance that patients required with handling the tablet was rated, and the time they took for data entry was recorded immediately after each assessment.

Data were entered via Apple iPads, using the Mobile Patient Survey [31], a web-based multilanguage electronic patient-reported outcome system. The standardized data processing and the standardized data export were realized with the single-source metadata architecture transformation [32], an extension of the electronic health record system which uses Module Driven Software Development to generate standardized apps.

Completed digital assessments were exported into the electronic health record automatically and could be accessed in full detail (including patient responses to all questions or items) by the clinical team. Participants did not receive insight into their data automatically, however, clinicians could provide them with updates on individual outcomes or assessment results upon request.

### Assessments and Measures

Reasons for exclusion were predefined according to the following categories: organizational reasons, severe cognitive deficits, insufficient language skills, and objective mental distress, with the last item referring to any psychological symptoms that would hinder participation or the ability to consent. When eligible participants refused, their reasons for refusal were recorded and later classified into 4 categories: lack of interest in the study, subjective mental distress, lack of general adherence, and data security concerns.

An external judgement of patients’ tablet-handling competency was made at each assessment based on a 4-point Likert-scale according to the following categories: 1, no required support: patient enters data independently; 2, little required support: patient needs few instructions before entering data; 3, some required support: patient needs instructions several times during data entry; and 4, a lot of required support: patient largely depends on the researcher for data entry. The median was calculated from all support ratings.

The researcher kept the time in minutes of each data entry. In order to achieve an individual entry pace factor, which signifies the deviation from the group mean, patients’ individual times for the baseline, interim, and discharge assessments were divided by the group mean for each assessment. A mean was calculated from these 3 assessments, resulting in a relational measure of individual data entry pace.

A digital version of the Beck Depression Inventory (BDI) [33,34] was used as a self-report measure of depressive symptoms. The Hamilton Depression Scale (HAMD) [35] and the Global Assessment of Functioning (GAF) [36] were conducted by the researcher as an objective measure of depression severity and global (ie, psychological, social, and occupational) functioning. An overview of all instruments included in the assessment battery can be found in Multimedia Appendix 1.

### Statistical Analyses

Statistics were computed using SPSS software (version 26; IBM Corp). For all models, uncorrected *P* values as well as Benjamini-Hochberg false discovery rate–corrected *P* values were generated.

### Participants

#### Required Support

To assess the influence of age, gender, depression severity and global functioning on the amount of required researcher support during data entry, we estimated an ordinal logistic regression model that included age, gender, and the baseline sum scores for BDI, HAMD, and GAF as predictors and required support as the dependent variable.

#### Data Entry Pace

A linear regression model was used to investigate the influence of these same variables on data entry pace. We estimated a linear regression model with age, gender, and the baseline sum scores for BDI, HAMD, and GAF as predictors and entry pace as the dependent variable.

#### Self-Report Measure Validation

In order to validate the self-report measure of depression severity with an external assessment, BDI and HAMD baseline sum scores were first correlated. To check for differences in agreement between self-reports and external assessments depending on age and gender, we additionally investigated potential interactions with age and gender based on linear regression models. The first model included BDI, age, and the interaction term age × BDI as predictors and HAMD as the dependent variable. The second model included BDI, gender, and the interaction term gender×BDI as predictors and HAMD as the dependent variable.

### Nonparticipants

Two-tailed independent sample *t* tests and chi-square tests were calculated to assess whether patients who were excluded by clinicians or study personnel and patients who refused participation differed in age or gender. The same tests were used to assess age and gender differences between participants and nonparticipants, while potential differences in depression severity and global functioning between these 2 groups could not be compared, as the data were not available for nonparticipants.

## Results

### Participants

#### Overview

All 242 participants were diagnosed with an affective disorder. The majority of our sample had a diagnosis of major depressive disorder (215/242, 88.8%), and 11.2% (27/242) had bipolar disorder. Additionally, 97 out of 242 participants (40.1%) were diagnosed with at least 1 psychiatric comorbidity, such as anxiety disorders, eating disorders, or personality disorders while 40 out of 242 (16.5%) participants also had a diagnosed somatic comorbidity. On average, participants completed 4 assessments during their hospital stay with a minimum of 1 and a maximum of 15 assessments. The average length of stay was 9.46 weeks (SD 6.88). Adherence with assessments was generally high, with an average of 79% of expected assessments being completed during the duration of treatment. Two participants dropped out of the study before their scheduled discharge from inpatient treatment, stating that the regular participation was disrupting their daily schedule. Two additional participants had to be excluded after initial participation: 1 due to cognitive limitations exacerbated by electroconvulsive therapy and 1 due to the development of delirious symptoms during the treatment course.

#### Required Support

Of 242 participants, 216 (89.3%) participants did not require support and managed data entry independently during all assessments. Little support was needed by 16 patients (6.6%), whereas 7 patients (2.9%) required some support, and 2 patients (0.8%) struggled to enter data independently and relied largely on the researcher for assistance.

For the ordinal logistic regression, predictor variables were tested a priori to rule out violations of the assumption of multicollinearity. Model fit was given (χ_5_^2^=26.1, *P<*.001). According to Nagelkerke *R*^2^, the model explained 23.1% of the variance in required support. Age was found to be the only significant contributor to the model as can be seen in Table 1; the odds of needing support with data entry and tablet handling increased with older age (odds ratio [OR] 1.08, 95% CI 1.04-1.12; *P*_FDR_=.004). Gender (*P*_FDR_=.94), depressive symptom severity (BDI: *P*_FDR_=.68; HAMD: *P*_FDR_=.52), and global level of functioning (*P*_FDR_=.34) did not contribute significantly to the model.

**Table 1:** Ordinal logistic regression analysis results predicting the effects of age, gender, and symptom severity on support required during data entry.

Variables^a^	B (SE)	Wald	Odds ratio (95% CI)	*P* value	*P*_FDR_ value^b^
Age	0.08 (0.02)	15.96	1.08 (1.04-1.12)	<.001	.004
Gender (male=1)	0.16 (0.54)	0.09	1.18 (0.41-3.42)	.76	.94
Beck Depression Inventory	–0.03 (0.04)	0.69	0.97 (0.90-1.05)	.41	.68
Hamilton Depression Scale	–0.08 (0.07)	1.15	0.93 (0.81-1.07)	.28	.52
Global Assessment of Functioning	–0.07 (0.05)	2.03	0.93 (0.85-1.03)	.16	.34

^a^Model Nagelkerke *R^2^*=0.231.

^b^Benjamini-Hochberg false discovery rate–corrected *P* value.

#### Data Entry Pace

It took participants 42.6 minutes (SD 16.6) on average to complete the baseline assessment, 7.0 minutes (SD 3.1) for each interim assessment and 18.3 minutes (SD 5.7) for the final assessment upon the conclusion of their treatment.

The linear regression model was significant and explained 31.1% of the variance in data entry time (*R*^2^=0.311, *F*_5,213_=19.0, *P<*.001). Age was a significant predictor of entry pace (β=0.519, *t*=8.90, *P<*.001, *P*_FDR_=.004). There was a trend for global level of functioning to predict entry pace, although this association was not upheld when controlling for multiple comparisons (β=–0.184, *t*=–2.24, *P*=.03, *P*_FDR_=.09). Gender (*P*_FDR_=.27) and level of depressive symptoms (BDI: *P*_FDR_=.94; HAMD: *P*_FDR_=.94) revealed no effect (Table 2).

**Table 2:** Linear regression results predicting the effects of age, gender, and symptom severity on required time for data entry.

Variables^a^	B (SE)	β	*t* value	*P* value	*P*_FDR_ value^b^
Age	0.009 (0.001)	0.519	8.90	<.001	.004
Gender	–0.053 (0.033)	–0.095	–1.61	.11	.27
Beck Depression Inventory	0.000 (0.002)	0.019	0.233	.82	.94
Hamilton Depression Scale	–0.001 (0.004)	–0.012	–0.13	.90	.94
Global Assessment of Functioning	–0.006 (0.003)	–0.184	–2.24	.03	.09

^a^Model *R*^2^=0.313.

^b^Benjamini-Hochberg false discovery rate–corrected *P* value.

#### Self-Report Measure Validation

We found overall high agreement between the patient reported outcome of depression severity and the external clinical rating of depression severity as demonstrated through a strong positive correlation between BDI and HAMD sum scores (*r*_214_=0.69, *P<*.001). The additional regression models confirmed these results: The first regression model was significant, with BDI and gender explaining 47.3% of the variance in HAMD (*F*_3,212_=63.4, *P<*.001, *R*^2^=0.473). BDI was a significant predictor (*F*_1,212_=186.3, *P<*.001, *P*_FDR_=.004) while gender was not (*F*_1,212_=0.3, *P*=.58, *P*_FDR_=.90). There was no significant interaction between BDI and gender (*F*_1,212_=0.1, *P=*.717, *P*_FDR_=.94). The second regression model was also significant, with BDI and age explaining 48.2% of the variance in HAMD (*F*_3,212_=65.9, *P<*.001, *R*^2^=0.482). BDI was a significant predictor (*F*_1,212_=18.2, *P<*.001, *P*_FDR_=.004) while age was not (*F*_1,212_=0.1, *P*=.81, *P*_FDR_=.94). There was no significant interaction between BDI and age (*F*=1.3, *P*=.26, *P*_FDR_=.52).

### Nonparticipants

Out of the 364 patients who were eligible for inclusion, 122 (33.5%) were excluded or refused to participate. The group of nonparticipants could be split into patients who were excluded by clinicians or study personnel (77/122; 63.1%) and patients who refused participation upon being approached for the study (45/122; 36.9%). There were no differences in age (t_120_=0.207, *P*=.84, *P*_FDR_=.94) or gender (n=122, χ_1_^2^=0.003, *P*=.96, *P*_FDR_=.96) between these 2 subgroups.

**Figure 1:**
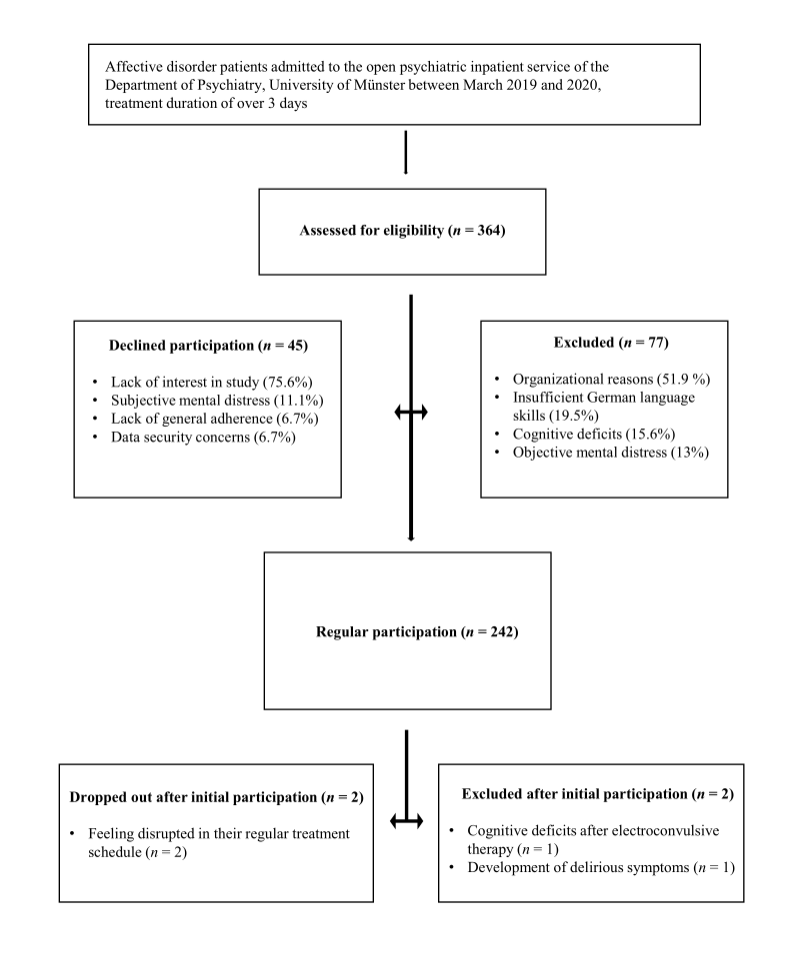
Study flow chart.

Half of the patients who had to be excluded from the study were excluded due to organizational reasons such as a very short hospital stay (ie, under 1 week). Other reasons for exclusions were insufficient German language proficiency, limited cognitive ability (ie, severe attentional or memory deficits), and acute mental distress as judged by the treating clinician (ie, severe agitation, psychotic symptoms, or tendency to dissociate). Within the group of patients who refused to participate, a majority cited general noninterest in the study as their reason for refusal as they were not willing to take on the extra effort of completing regular assessments during the course of treatment. Some patients also expressed that they were not interested, as they did not feel like they would personally benefit from the study. Fewer patients expressed that they felt too incapacitated by their symptoms to participate, displayed general nonadherence with treatment and thus refused to participate in additional assessments, or expressed concerns over data security. Please refer to Figure 1 for more detailed visualization of the distribution of reasons for exclusion and refusal to participate.

The nonparticipating group was significantly older (t_362_=3.31, *P<*.001, *P*_FDR_=.004), and there was a trend of this group to consist of more women than the participating group, although this association was not upheld when correcting for multiple comparisons (n=364; χ_1_^2^=4.34, *P*=.04, *P*_FDR_=.11). See Table 3 for sociodemographic and clinical characteristics.

**Table 3:** Sociodemographic and clinical characteristics of patients who were initially approached for participation, consisting of 122 nonparticipating patients and 242 study participants.

Variables		Nonparticipants (n=122)	Participants (n=242)	*P* value	*P*_FDR_ value^a^
**Age (years)**		48.52		<.001	.005
	Mean (SD)	48.52 (19.00)	41.93 (17.39)		
	Range	18-89	18-81		
**Gender, n (%)**				.04	.11
	Male	43 (35.2)	113 (46.7)		
	Female	79 (64.8)	129 (53.3)		
**Beck Depression Inventory**				—^b^	—
	Mean (SD)	N/A^c^	24.09 (11.24)		
	Range	N/A	1-50		
**Hamilton Depression Scale**				—	—
	Mean (SD)	N/A	15.53 (6.16)		
	Range	N/A	1-31		
**Global Assessment of Functioning**				—	—
	Mean (SD)	N/A	57.44 (8.63)		
	Range	N/A	33-78		

^a^Benjamini-Hochberg false discovery rate–corrected *P* value.

^b^Statistical test not performed.

^c^N/A: not applicable.

## Discussion

With this study, we demonstrated the feasibility and acceptance of repeated digitized assessment of risk and symptom profiles in affective disorder inpatients. Our results indicate that participatory medicine can be achieved in patients with affective disorders, as they are willing and able to contribute self-report measures throughout the duration of their inpatient treatment. This study, therefore, provides important insight into the possibility of routinely collecting longitudinal data in real-world clinical cohorts that may guide the way toward personalized psychiatric care.

During an assessment period of 1 year, we achieved an inclusion rate of 66.5% of patients with a diagnosed affective disorder. This rate is similar to those reported from other investigations performed on the general population and nonpsychiatric patient groups, which indicates that major depressive disorder or bipolar disorder symptomology does not constitute a barrier toward participation [37,38]. Adherence to assessments was high, and the dropout rate after initial participation was very low. Exclusion by the clinician or researcher and refusal to participate by the patients themselves were largely not due to symptom severity or cognitive impairment but for organizational reasons or a general disinterest in the study, which mirrors reasons for nonparticipation in research from nonpsychiatric populations [39]. It also confirms previous research on the generally high level of acceptance of longitudinal self-reporting technology in patients with bipolar disorder [40,41].

A vast majority of patients who did participate were able to enter data independently and did not encounter technical difficulties. More importantly, we found no association between symptom severity at baseline and the amount of required support in handling the equipment or prolonged data entry times. This is in line with previous investigations on the feasibility and acceptability of digitally based assessments in psychiatric populations [29]. Moreover, we were able to demonstrate the validity of patient reports with the use of an external measure of depression severity performed by a clinical interviewer. The level of agreement between self-reported depression severity with the BDI and the external rating based on the HAMD was comparable to findings from the literature [42] and indicated high validity of the digital self-report measure. This is an especially promising result for the implementation of patient-reported data collection technologies into routine documentation as well as its use for research purposes because it indicates that little to no additional personnel resources are required in order to gain valuable longitudinal data throughout the course of treatment. Moreover, the digital implementation of such assessments will allow for more accurate data collection and immediate data storage and analysis [15]. In the future, such an infrastructure of digital data collection could be used to communicate treatment outcomes and visual representations thereof directly to patients. Similar approaches have been found to improve communication between patients and health care providers [43] and would also constitute an improvement in the participatory aspect of precision medicine.

Although our results generally support the feasibility of longitudinal digital data collection in affective disorders, a few systematic recruitment and accessibility issues must be addressed. Despite the fact that no association between symptom severity and performance during data entry in our participating sample was detected, 6% of all potential participants were excluded beforehand due to reduced cognitive ability or clinicians’ concerns over their acute mental distress. Although this embodies only a small percentage of our sample, this result suggests that a systematic exclusion of more severe cases may not be avoidable. Nevertheless, it may be worthwhile to critically consider such cases individually, as it has been shown that carers overestimate the amount of distress patients are put under during research participation [24].

We, furthermore, found a statistical trend for women to be more likely to refuse or be excluded from the study than men. Although this finding did not survive the correction for multiple comparisons, it is nevertheless meaningful to consider potential reasons this gender disparity may occur, as it may again be due to symptom severity. Although the reasons recorded at the time of exclusion do not suggest symptom severity is the main factor, evidence does suggest that women are more likely to seek psychiatric treatment and report more severe symptoms [44,45]. As we do not have data on the symptom levels of the excluded group, this question cannot be answered with certainty.

Regardless of gender, the factor that impacted both the amount of required assistance and time during data entry was older age. Older adults found it more difficult to handle the tablet-based assessments and took longer to complete them. However, the percentage of participants who needed assistance was comparatively small, and even those who did require assistance were able to complete assessments regularly, which suggests that their difficulties with handling the equipment did not stop them from participating. Although our study did not assess subjective attitudes toward technology, previous studies [38,46] found that digital methods of data collection are well-accepted even among older adults. It can also be expected that technological literacy will rise in older populations over the years, as smartphones and tablets are becoming increasingly ubiquitous, which will alleviate the difficulties for this specific age group in the future. Studies [47,48] also show that, although older adults lag behind in digital literacy, such competencies can be acquired through social support. Nevertheless, our findings suggest that options for support of older participants should currently be offered to not systematically exclude technologically less well-versed patients from participatory care. 

In addition to older age, there was also a trend for lower global functioning to be related to slower data entry pace; however, it was not associated with the amount of required assistance. Even though this association was not upheld when correcting for multiple comparisons, this finding should also be critically discussed. It suggests that patients with a generally lower level of global functioning take longer to complete assessments but are still able to do so independently. Moreover, the added time expenditure does not lead to participants dropping out of the study, indicating that the slower entry pace is tolerable and not a barrier that would keep lower functioning patients from participating in such research.

Furthermore, patients who are not proficient in the language spoken by their health care providers are a systematically disadvantaged group in psychiatric care who could not be included in this investigation. At equal or greater levels of need, persons with an immigration background are known to seek mental health treatment less often and are less likely to report favorable treatment outcomes [49,50]. This suggests that the inclusion of marginalized populations would be of great importance especially when investigating individual risk factors for affective disorders on the way to precision psychiatry. In fact, digitally assessed self-report measures present the opportunity to get detailed, standardized assessments despite language barriers, as questionnaire measures can be made available in every language. The app that we used for data collection supports the implementation of multilanguage assessments [51] and could therefore be used in future investigations in order to also reach and assess non-German speaking clinical populations. This would provide a wealth of standardized, quantifiable information about patients of diverse cultural backgrounds that could guide treatment but also assist in identifying suitable interventions for clinical populations with specific ethnic or cultural differences and risk or resilience factors.

To the best of our knowledge, this study is the first naturalistic investigation to incorporate repeated, digitally assessed patient reports throughout the course of inpatient treatment in affective disorders. Overall, the acceptability and feasibility of such study protocols within the clinical routine is high while required resources remain comparatively low. Patients are willing and able to provide data at regular intervals and are not systematically disadvantaged by the severity of their affective symptoms. Future implementations should keep gender, age, and cultural factors in mind when approaching patients and offer assistance with any technological equipment as needed.

In conclusion, this study is a first step in demonstrating that the participatory aspect of precision medicine can be achieved in psychiatry. In the future, the information gathered routinely through patient reported assessments could be combined with other potential data sources such as fitness trackers and information gained from electronic health records [25,52-54]. This may pave the way for data-driven predictive models that could, in the more distant future, be used to predict and prevent the occurrence of affective disorders, as well as facilitate the identification of individual risk profiles. Even without the use of predictive modeling, self-reports and the direct exchange of such information between patients and clinicians might improve treatment [43]. Overall, such advances in psychiatry will be invaluable as personalized treatments tailored to such individual risk factors may lead to much shorter and less frequent hospitalizations, which would equate to more cost-effective treatments and a pronounced reduction in patient suffering.

## Appendix

Multimedia Appendix 1 Supplementary table.

## Abbreviations

BDI: Beck Depression Inventory
GAF: Global Assessment of Functioning
HAMD: Hamilton Depression Scale
