# Supplementary material for: Repeated Digitized Assessment of Risk and Symptom Profiles During Inpatient Treatment of Affective Disorder: Observational Study
Source: JMIR Ment Health. 2020 Dec 1;7(12):e24066. doi: 10.2196/24066 (PMC7738257; doi:10.2196/24066)
Supplement: Multimedia Appendix 1 [file mental_v7i12e24066_app1.docx]

## Supplementary material

*Table S1* Overview of questionnaires and number of items included in each assessment battery.

| *Self-report measures* | *Baseline assessment* | *Interim assessment* | *Pre-discharge assessment* | *Number of items* |
| --- | --- | --- | --- | --- |
|  |  |  |  |  |
| Sociodemographic questionnaire | X |  |  | 5 |
| Family mental health history | X |  |  | 11 |
| Questions on individual disease course | X |  |  | 14 |
| BDI | X | X | X | 21 |
| CTQ | X |  |  | 28 |
| Questions on somatic comorbidities | X |  |  | 5 |
| SCL-90 Somatization Scale | X |  | X | 12 |
| BFI-2-S | X |  | X | 30 |
| NARQ-S | X |  | X | 6 |
| BFI-2-XS |  | X |  | 15 |
| *External assessments* |  |  |  |  |
|  |  |  |  |  |
| GAF | X |  | X | 1 |
| HAMD | X |  | X | 22 |

*Abbreviations*. BDI, Beck’s Depression Inventory [1], [2]; BFI, Big Five Inventory [3]; CTQ, Childhood Trauma Questionnaire [4]; GAF, Global Assessment of Functioning [5]; HAMD, Hamilton Depression Scale [6]; NARQ-S, Narcissistic Admiration and Rivalry Questionnaire [7]; SCL, Symptom Checklist [8].

## References

[1] A. T. Beck, R. A. Steer, and G. K. Brown, *Beck Depression Inventory (BDI-II)*. Pearson, 1996.

[2] M. Hautzinger, M. Bailer, H. Worall, and F. Keller, “Beck-Depressions-Inventar (BDI),” *Bern Huber*, 1994.

[3] C. J. Soto and O. P. John, “Short and extra-short forms of the Big Five Inventory–2: The BFI-2-S and BFI-2-XS,” *J. Res. Pers.*, vol. 68, pp. 69–81, 2017, doi: 10.1016/j.jrp.2017.02.004.

[4] D. P. Bernstein, L. Fink, L. Handelsman, and J. Foote, “Childhood Trauma Questionnaire,” *Assess. Fam. violence A Handb. Res. Pract.*, 1998, doi: https://doi.org/10.1037/t02080-000.

[5] R. C. W. Hall, “Global Assessment of Functioning: A Modified Scale,” *Psychosomatics*, vol. 36, no. 3, pp. 267–275, 1995, doi: 10.1016/S0033-3182(95)71666-8.

[6] M. Hamilton, “The Hamilton Rating Scale for Depression,” in *Assessment of Depression*, Springer, 1986, pp. 143–152.

[7] M. Leckelt *et al.*, “Validation of the narcissistic admiration and rivalry questionnaire short scale (NARQ-S) in convenience and representative samples,” *Psychol. Assess.*, vol. 30, no. 1, pp. 86–96, 2018, doi: 10.1037/pas0000433.

[8] L. R. Derogatis and K. L. Savitz, “The SCL-90-R, Brief Symptom Inventory, and Matching Clinical Rating Scales.,” 1999, doi: https://doi.org/10.1037/t02080-000.
